# Effect of Salt (Sodium Chloride) Replacement With Potassium Chloride, High Pressure Processing, and Cold Storage at 4°C on Beef Sausage Volatile Compounds

**DOI:** 10.3389/fnut.2022.856089

**Published:** 2022-04-14

**Authors:** Theodora Ojangba, Li Zhang, Solomon Boamah, Yanlei Gao, Zhuo Wang, Martha Wunnam Alhassan

**Affiliations:** ^1^College of Food Science and Engineering, Gansu Agricultural University, Lanzhou, China; ^2^Department of Food Science and Technology, University for Development Studies, Tamale, Ghana

**Keywords:** beef sausage, salt-replacement, high-pressure processing, lipid oxidation, volatile compounds

## Abstract

This study aimed to investigate the partial substitution of 0, 25 and 50% sodium chloride (NaCl) by potassium chloride (KCl) coupled with high-pressure processing (HPP) effects on volatile compounds and lipid oxidation of beef sausage at five (0, 3, 7, 14, and 21) different cold storage days (4°C). The odor activity values (OAV) of the various compounds were visualized by heat map analysis. Thiobarbituric acid reactive substances (TBARS) of the samples treated with 100% NaCl and HPP increased by an average of 0.52 ± 0.01 mg MDA/kg compared with the control (100% NaCl-no HPP) across the 21 storage days. In addition, 50% NaCl substitution with KCl in combinations with HPP treatments increased TBARS across the 21 storage days by an average of 0.40 ± 0.02 mg MDA/kg compared with no HPP treatment. However, on day 3, there was a sharp decrease in TBARS by an average of 0.10 ± 0.01 mg MDA/kg compared with days 0, 7, 14, and 21 in all treatments. At the end of 21 days of storage, a total of 227 volatile compounds were identified and quantified in the beef sausage, including 43 aldehydes, 46 phenols, 8 ketones, 30 alcohols, 14 acids, 12 esters, 27 terpenes, and 47 alkanes. However, no ketone compounds were detected on days 7, 14 and 21; esters on day 14 and acids on days 14 and 21 in the samples treated with or without HPP across the salts levels. However, high OAVs (OAV > 1) were obtained after partial substitution of NaCl with KCl at 25 and 50% with HPP treatment compared to the samples not treated with HPP. The aroma perceived in the beef sausage was due to compounds with the highest OAVs such as; pentadecanal, benzyl carbazate, anethole, myristicin, o-cresol, phenylacetaldehyde and (E)-methyl isoeugenol, pentadecanal, hexanoic acid, octanoic acid, eugenol, trans-2-nonenal, trans-2-octenal, trans-2-decenal, 2-butyl-1-octanol, 2,3-butanedione, ethyl hexanoate, ethyl octanoate, (-)-4-terpineol which had an OAV > 1 as compared to the other compounds with an OAV < 1. In conclusion, 25 and 50% NaCl partial replacement with KCl coupled with HPP technique can be considered in producing low-NaCl beef sausage in order to improve the flavor and decrease lipid oxidation during cold storage.

## Introduction

Low-salt beef sausages are highly appreciated in many countries for their mildly sour taste. The production of these products is based on the use of low temperatures during maturation, which avoids intense fermentation and a strong sour taste (1).

In meat products, sodium chloride (NaCl) serves many purposes. The qualitative properties of meat products are improved by NaCl. First, NaCl increases the ability of meat products to bind and retain water, which leads to the production of the desired gel texture (2). Secondly, NaCl can promote the development of flavors and salty taste characteristics by controlling important biochemical and enzymatic reactions. NaCl has a salting-out effect and can increase the release of flavor compounds from the food matrix (3). In addition, NaCl contributes to a reduction in water activity, which is an important parameter for controlling the growth of harmful bacteria and extending the shelf life of meat products (4).

Foods, particularly fermented meat products, account for about 20–30% of salt consumption in a person’s diet, according to some research (5, 6). Water migration and evaporation can cause NaCl content to rise to 3.0–5.0% during prolonged fermentation (7). Excessive NaCl consumption has also been linked to a higher risk of hypertension, stroke, and vascular disease (8). Beef sausages are a popular cooked meat product all over the world, making them a perfect target for lowering salt consumption among the general population. Direct NaCl reduction and the use of salt substitutes to replace NaCl are the two most common strategies for reducing NaCl level in fermented meat products (9, 10). Several studies (11, 12) have indicated that replacing sodium chloride (NaCl) in foods such as beef sausages benefits both humans and the economy (13).

High-pressure processing (HPP) is a non-thermal method for inactivating/killing spoilage and dangerous bacteria while preserving the qualities of minimally processed foods (14). In the meat processing industry, HPP is a good alternative to thermal pasteurization after production (14) for preventing post-processing contamination. HPP’s effects on shelf life, microbiological safety, and quality of low-salt beef sausages and cooked ham have been studied (15, 16).

Food odor is linked to the volatile molecules released by food and can be used to assess its quality and safety. Simultaneous distillation and extraction (SDE) (17) and solid-phase microextraction (SPME) (18) have both been employed previously. There is no one-size-fits-all solution for isolating volatile chemicals from food. In addition, each method is subject to error in the extraction of compounds (19), resulting in disparities in volatile profiles of the same product (20). Given the increased popularity of beef sausages, it’s important to see if the sodium content can be lowered without using salt alternatives (21). Some studies have looked at the effects of direct salt reduction in various meat products, such as bacon, ham, and salami, and concluded that salt content can be reduced without affecting general acceptance (8, 22). However, there are fewer studies on the effects of partial replacement of NaCl by KCl in combinations with HPP on volatile compositions and lipid oxidation in beef sausage during cold storage. As a result, a new study may be required to investigate the effects of partial substitution of 25 and 50% NaCl by KCl in combinations with HPP (100 MPa for 5 min at 25°C) on the volatile compounds and lipid oxidation in beef sausage at five (0, 3, 7, 14, and 21) cold storage days (4°C). We hypothesize that HPP approaches may be used to characterize the volatile compounds and lipid oxidation in beef sausage salted with partial replacement of NaCl by KCl, thereby providing novel insights into the flavor quality and cold storage effect of low sodium beef sausage, and provide guidance for future research.

## Materials and Methods

### Experimental Design for Reduced-Sodium Chloride Beef Sausage

The extreme vertices mixture design was used to create the two-component salt combination (NaCl and KCl) in each beef sausage formulation according to Anderson and McLean (23). In summary, the salt mixtures were generated by Design-Expert, a statistical package (Statease Inc., Minneapolis, MN, United States). Each salt mixture was employed to substitute NaCl (a sum of 100 percent in an experimental mixture design) of the actual beef sausage as described in Table 1. The study was designed in two stages: in the first stage, different levels of salt replacement were used to select the best salt level based on several preliminary sensory tests (data not provided). Minced beef samples were randomly divided into three groups before the salting stage. Treatments were as follows: control (100% of NaCl), treatment 1 (75% NaCl and 25% KCl) and treatment 2 (50% NaCl and 50% KCl). In the second stage, each treatment group was further divided into two; each part was subjected to or without high-pressure processing at 100 MPa for 5 min at 25°C. In all, six (6) treatment groups of sausages were formulated. Three batches were prepared for each treatment group, with each containing 35 chubs of beef sausages (length 10 cm). The experimental ranges for these two salt components were established following the amount recommended by the Chinese Dietary Guidelines (24) (<6 g per day).

**TABLE 1:** Percentages of sodium chloride and potassium chloride used in the formulation of beef sausage.

First stage			
Ingredient	Treatments (%)		
	Control	T1	T2
Beef lean meat	75	75	75
Beef back fat	12.39	12.39	12.39
Water (cold ice)	10	10	10
Spices	0.4	0.4	0.4
Phosphate	0.2	0.2	0.2
NaNO_2_	0.01	0.01	0.01
Sodium chloride (NaCl)	2	1.5	1
Potassium chloride (KCl)	–	0.5	1
Total	100	100	100
**Second stage**			
HPP	100% NaCl	75% NaCl and 25% KCl	50% NaCl and 50% KCl
No HPP	100% NaCl	75% NaCl and 25% KCl	50% NaCl and 50% KCl

*Control (100% NaCl- No HPP); T1 (75% NaCl and 25% KCl-No HPP); T2 (50% NaCl and 50% KCl-No HPP) and control (100% NaCl-HPP); T1 (75% NaCl and 25% KCl-HPP); T2 (50% NaCl and 50% KCl-HPP).*

### Reduced-Sodium Chloride Beef Sausage Preparation

Six kilograms (6 kg) of fresh beef (semimembranosus muscles) and 1 kg beef back fat was purchased from a local BHG supermarket (Lanzhou, China). Fresh beef and fat were vacuum-packed and stored at -18°C until required for sausage production. The frozen meat and fat was allowed to thaw slightly before being minced. The meat was trimmed of excess fat, minced through a 5-mm plate using a vacuum mincer (BX150, Hengshun Machinery Factory, Shandong, China) at 75 rpm for 3 min. All salts including (NaCl and KCl) were food grade salts purchased from Awell Ingredients Co., Ltd. Anhui, China. Phosphate salt (TTSPP) was purchased from Jiangsu Kolod Food Ingredients Co., Ltd., Jiangsu, China. Sodium nitrate (NaNO_2_) was purchased from Weifang Boteng Chemical Co., Ltd., Shandong, China. All other spices were purchased from a local BHG supermarket (Lanzhou, China). Mechanically grinded beef batter was placed into a bowl mixer (Mado, model MTK 662, Germany), mixed with NaCl and KCl salts only and half of the ice, and comminuted for 3–4 min at low speed for thorough mixing. Meat batters were then vacuum-packed (Djpack, DZ-450A, Wenzhou Dajiang Machine) in vacuum pouches of about 150 g in weight. Application of HPP was done according to Yang et al. (25) with modifications. Water was used as the pressure-transfer medium and the entire system was cooled to an initial ambient temperature of 25°C by a thermos-stated jacket. Samples for HPP were subjected to a pressure level at 100 MPa for 5min in an isotactic press (HPP. L2-600/1, Tianjin Huatai-Senmiao Bioengineering Technology Co., Ltd.) using 850 Mini FoodLab high-pressure vessels with a capacity of 0.3 l (Stansted Fluid Power Ltd., United Kingdom).

**FIGURE 1:**
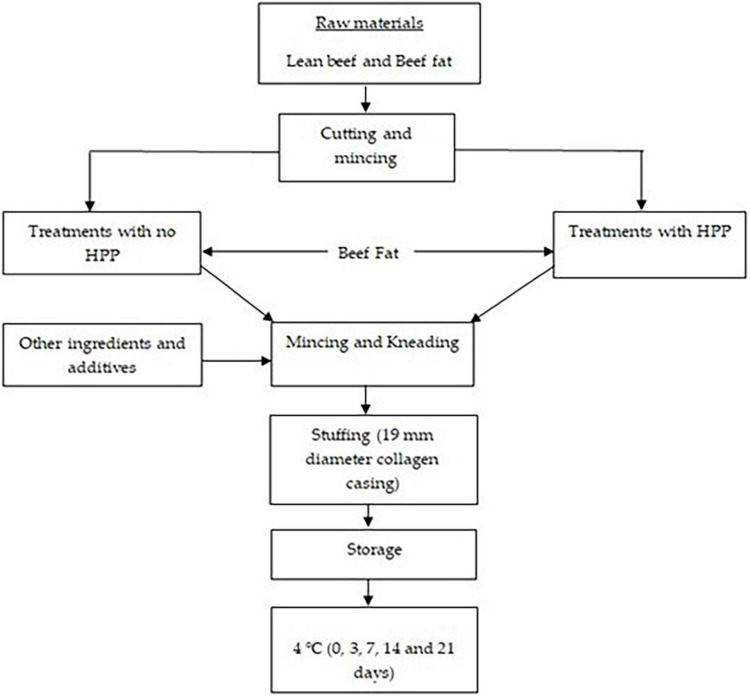
Flow diagram of the manufacture and storage of beef sausage under study (treatments with HPP and treatment with no HPP).

After HP treatment, the beef fat emulsion and the remaining ingredients were then added and emulsified at knife and bowl chopper (Hebei Yuanchang Food Mechanism and Technology Co., Ltd., China) at a speed of 2,780 rpm/24 rpm until the temperature reached 12°C. Each treatment batch was stuffed into pre-soaked collagen casings (19 mm diameter, UniPac, Edmonton, AB) and the casings were stretched and sealed. The six (6) treatment groups includes; control (100% NaCl- No HPP); T1 (75 NaCl and 25% KCl-No HPP); T2 (50 NaCl and 50% KCl-No HPP) and control (100% NaCl-HPP); T1 (75 NaCl and 25% KCl-HPP); T2 (50 NaCl and 50% KCl-HPP) with each group containing 35 chubs (length 10 cm). After preparation, 1/3 of beef sausages from each treatment were used for analysis, while the remaining 2/3 were vacuum-packed and stored 4°C for 0, 3, 7, 14, and 21 days in the absence of light. Three replications of the experiment were conducted on different days (Figure 1).

### Lipid Oxidation (Thiobarbituric Acid Reaction Substance) Analysis

Lipid oxidation was measured using the 2-thiobarbituric acid reactive substances (TBARS) assay (26) modified for interference with nitrites in cured meat (27). Briefly, 2.5 g of sausage sample was mixed with 10 mL of reverse osmosis water (RO) and 1.0 mL of sulfanilamide reagent [0.5% sulfanilamide in 20% HCl (v/v)] in a Magic Bullet blender and ground for 30 s. The mixture was then mixed with the sulfanilamide reagent. The mixture was placed in a round-bottomed boiling flask along with 10.0 mL of RO water used to wash the mixer. 2.0 mL of 4 N HCl was added to bring the mixture to a pH of 1.5. Glass beads (6) were added to the boiling flask, which was then brought to a vigorous boil on a hot plate set at 375°C (Corning PC –620D, Corning Incorporated Life Sciences, Tewksbury, MA). A simple glass distillation apparatus (hot plate, boiling flask, short vertical column connected to an inclined horizontal water-cooled condenser) was used to collect ∼10 mL of the distillate. 5.0 mL of the distillate was mixed with 5.0 mL of TBA reagent (0.02 M 2-thiobarbituric acid in 90% glacial acetic acid) in a 10 mL screw-capped glass tube and vortexed for 10 s at maximum speed (VWR Vortexer 2, VWR International Co., Edmonton, AB). The tubes were then immersed in a boiling water bath for 35 min and then cooled in tap water for 10 min. The absorbance at 532 nm of the resulting solution was measured using a spectrophotometer (00080S, Full wavelength microplate reader, China).

TBARS values were determined against a standard linear curve of malonaldehyde standard solution (1,1,3,3-tetra-ethoxypropane). TBARS values were expressed as milligrams malonaldehyde equivalents/kilogram sausage sample. The peak regions of six calibration standards were plotted against concentration. For the six concentration levels, each injected three times, the linear regression equation was: *y* = 0.0027× + 0.0004, where y is the peak area and x is the MDA concentration (ng/mL). The R^2^ coefficient for regression was 0.9979, showing high linearity. The method’s linearity was assessed using the analytical curve, which revealed a linear interval of 21.5–1720 ng/mL.

### Volatile Compound Analysis

Volatile compounds in the beef sausages were extracted by solid-phase microextraction (SPME) and analyzed by gas chromatography/mass spectrometry (GC/MS) as described by Wen et al. (28) with slight modifications. To evaluate the contribution of these compounds to the flavor profile of the sausages, the odor activity value (OAV) was calculated by dividing the volatile compound content by their threshold values from the database obtained from https://www.vcf-online.nl/VcfCompoundSearch.cfm.

### Gas Chromatography-Mass Spectrometry

Volatile compounds were identified and quantified by the headspace/solid phase micro-extraction (HS-SPME) and gas chromatography coupled to mass spectrometry (GC-MS) according to (29) with slight modifications. A triple SPME fiber of DVB/CAR/PDMS 50/30 μm (conditioned at 270°C/30 min), was exposed for 1 h and 30 min at 40°C. In a 20 mL capped vial, 10 g of minced beef sausages, 2 g of NaCl and 5 μL of internal standard (8.82 ppm, 2-octanol) were added. The vial was tightly capped and equilibrated in a water bath at 90°C for 30 min. The volatile compounds were transferred to the GC injector and desorbed (260°C/5 min). The analysis was performed in a spitless mode (50 mL/min during 2 min) on a 6,890 N Agilent gas chromatograph coupled to a 5,973 N Agilent Mass Detector. The selected chromatography conditions were: A polar column (DB-WAX, 60 m × 0.25 mm id; 0.25 μm film thickness), helium as the carrier gas (1 mL/min), and oven temperature started at 40°C/3 min, ramped at 3°C/min to 150°C and up to 220°C (7°C/min for 5 min). The MS operated with an electron energy of 70 eV using the electron impact mode, the ion source temperature was 230°C, and the scanning was carried out from 45 to 550 amu. Compounds were identified by comparison with mass spectra and the linear retention index (RI) with spectral data from NBS75K and Wiley G 1035 libraries, and calculating linear retention indices (LRI) relative to a range of alkanes (C_1_–C_19_). The method was validated according to ICH Q_2_ (R_1_) (30) guidelines for various parameters such as precision, linearity, accuracy, solution stability, robustness, limit of detection, and quantification. Chromatographic performance obtained from compounds was found to be acceptable with a relative standard deviation of the retention times of less than 0.01% RSD. The sums of the abundances of up to four characteristic ions per compound were used for semi-quantitative determination. Abundances of volatile compounds were referenced to the internal standard (IS) (compound peak area multiplied by 10^3^ and divided by IS peak area). Finally, concentrations of volatile compounds were obtained and expressed as ng/g.

### Statistical Analysis

The profile analysis of volatile compounds and lipid oxidation were subjected to two-way ANOVA to evaluate the effect of salts formulation, HPP, storage time, and their interactions. These analyses were accomplished using SPSS Win 12.0 software (SPSS Inc., Chicago, IL) and presented as a mean ± standard error of means (SEM). Statistical significance was assigned at *P* < 0.05 in Duncan’s multiple range tests using two-way ANOVA.

## Results and Discussion

### Effect of Partial Substitution of Sodium Chloride Coupled With High Pressure Processing on Lipid Oxidation Marker Thiobarbituric Acid Reactive Substances of Beef Sausage Under Different Cold Storage (4°C) Days

**FIGURE 2:**
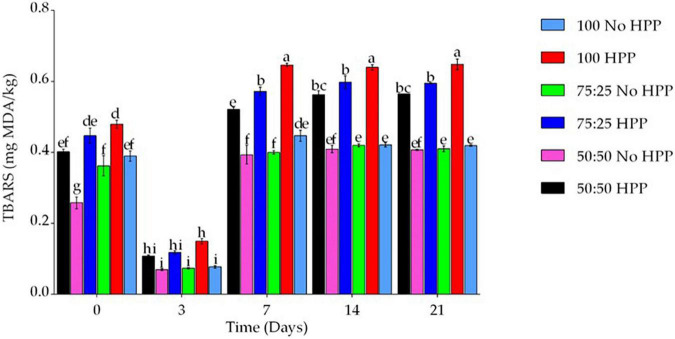
Effect of partial substitution of NaCl in combination with HPP on lipid oxidation marker Thiobarbituric Acid Reactive Substances (TBARS) of beef sausage under cold storage. Data were expressed as mean values and ± SEM. Different lowercase letters indicate significant differences at *P* < 0.05 in Duncan’s multiple range tests using two-way ANOVA.

Thiobarbituric acid reaction substance (TBARS) is mainly used to evaluate the degree of lipid secondary oxidation, which mainly reflects the amount of malondialdehyde compounds formed by lipid oxidation, so it is used to evaluate the degree of food oxidation and deterioration (31). In our study, partial substitution of NaCl with KCl in combinations with HPP significantly affected the TBARS across the storage days. The 100, 75 and 50% NaCl and coupled with HPP increased TBARS by an average of 0.52 ± 0.01, 0.46 ± 0.01 and 0.40 ± 0.02 mg MDA/kg compared with the 100, 75 and 50% NaCl without HPP across the 21 storage days (Figure 2). Again, an increase in the storage days increased the TBARS except day 3 where there was a sharp decrease in MDA content by an average of 0.10 ± 0.01 mg MDA/kg compared to days 0, 7, 14 and 21 in all the treatments. TBARS values reflected the degree of lipid oxidation (32, 33). The TBARS values brought out the trend which showed an initial rise and decreased later and showed insignificant changes during the NaCl replacement with KCl coupled with HPP at day 3. However, the maximum TBARS values appeared at the same time, possibly because malondialdehyde potentially react as a bifunctional group molecule with free amino acids and other small molecular components during processing (34, 35). As a result, the TBARS levels began to increase during the later storage days. Our findings are in agreement with those of Zhao et al. (36). who found that TBARS levels revealed a pattern that started out with an increase and then decreased with no significant changes after curing, fermentation, or drying of Chinese dry sausage with different NaCl content at 5 days of storage. Similar to these findings (37) reported decreases in TBARS due to the addition of KCl, and they argued that the replacement of NaCl with KCl in bacons treatments by more than 50% was higher compared with those with lower amounts. These findings are comparable to Zanardi et al. (38) in Italian salami and (39) in dry-cured ham who reported that the replacement of NaCl with KCl by more than 50% resulted in significantly increased TBARS values compared with control (100% NaCl).

### Effects of Partial Substitution of Sodium Chloride With Potassium Chloride and High Pressure Processing on Aldehyde, Alcohol and Ketone Compounds

**FIGURE 3:**
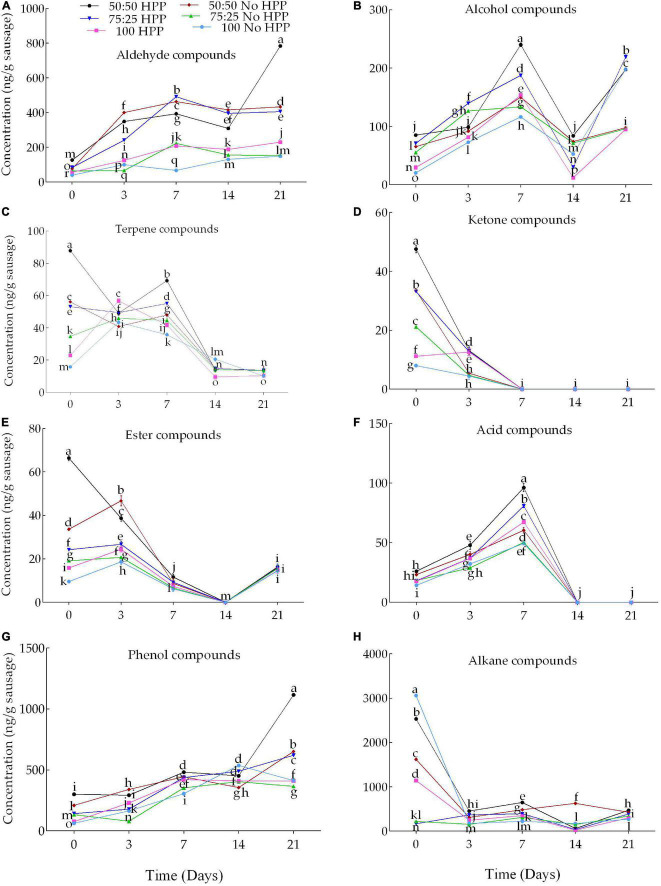
Effects of partial substitution of NaCl with KCl at 0 25 and 50% coupled with HPP on volatile compounds [**(A)** aldehyde, **(B)** alcohol, **(C)** terpene, **(D)** ketone, **(E)** ester, **(F)** acid, **(G)** phenol and **(H)** alkane] under five (0, 3, 7, 14, and 21) different cold storage days (4°C). HPP treated at 100 MPa for 5 min at 25°C. Bars indicate standard error of the means. At each of the storage times, different letters indicate significant (*P* < 0.05) differences between treatments.

Lipid oxidation and hydrolysis are the chief sources of flavor formation in sausages and produce more than 80% of volatile flavor compounds (36). While mild oxidation provides a desirable flavor during processing, excessive oxidation can result in unpleasant flavors such as rancidity and a nauseous odor, which can harm consumers’ health. In this current study, a total of 227 volatile compounds were identified and quantified in the beef sausages, including 43 aldehydes, 46 phenols, 8 ketones, 30 alcohols, 14 acids, 12 esters, 27 terpenes, and 47 alkanes at the 21 storage days. However, no ketone compounds were detected on days 7, 14 and 21; esters on day 14 and acids on days 14 and 21 in the samples treated with or without HPP across the salts levels. Our findings are similar to that of (40) who reported that 142 volatile compounds including acids, alcohols, aldehydes, benzenic compounds, esters, ethers, hydrocarbons, ketones, nitrogen compounds, sulfur compounds, terpenoids, and halogenated compound following HPP treatment in cured-cooked “lacón.” A total of 14, 12, 9, 4 and 4 aldehydes were detected on days 0, 3, 7, 14 and 21, respectively. An increase in the storage duration decreased the aldehyde and alcohol compounds. However, the compounds were significantly (*P* < 0.05) affected by the application of HPP and partial substitution of NaCl by KCl (Figure 3A). Compared to the control, the HPP treated beef sausages with partial substitution of NaCl with KCl at 50, 25 and 0% increased the concentration of aldehyde compounds by an average of 72.14, 65.42 and 36.46%, respectively across the 21 storage days. Similarly, a total number of 10, 8, 5, 4, 3 alcohols were identified on days 0, 3, 7, 14 and 21, respectively. Compared to the control, the HPP treated beef sausages with partial substitution of NaCl with KCl at 50, 25 and 0% increased the concentration of alcohol compounds by an average of 51.23, 52.32 and 22.05%, respectively across the 21 storage days (Figure 3B). Four (4) and 2 ketones were detected on days 0 and 3, respectively. Compared to the control, the HPP treated beef sausages with partial substitution of NaCl with KCl at 50, 25 and 0% increased the concentration of ketones compounds by an average of 74.45, 70.32 and 46.18%, respectively across the 0 and 3 storage days. However, no ketone compounds were detected on days 7, 14 and 21 in the samples treated with or without pressure with partial substitution of NaCl by KCl across the salts levels (Figure 3D). The unstable double bonds of compounds are easy to oxidize, and produce a series of oxidation products including aldehydes, ketones, and alcohols (41). Detecting the oxidation products at the primary and the secondary stage are the most common methods to evaluate the degree of lipid oxidation (31). Aldehydes are typical flavor compounds originating from oxidative (auto-oxidation) and thermal degradation of lipids, Strecker degradation of amino acids, and thiamine degradation (42–44). The content of these aldehydes increased from increased KCl substitution to increased storage time due to higher formation rate of lipid-derived compounds by autoxidation. During storage, the contents of these aldehydes mainly increased with significant differences observed between the days. This could be explained by the free fatty acid accumulation and lipid oxidation processes during storage. Our results had a similar trend with the findings of (45), who investigated volatile compounds in beef frankfurter production during 6-week storage. As a result, managing the degree of lipid oxidation is critical in the processing of sausages, as it not only ensures quality but also protects the health of consumers. Lipid hydrolysis has a promoting effect on lipid oxidation (46). Lipid oxidation and hydrolysis are common reactions in the processing of cured meat products (47).

### Effects of Partial Substitution of Sodium Chloride With Potassium Chloride and High Pressure Processing on Terpenoid, Ester and Acid Compounds

Three factors including formulation, HPP and storage time (and their interaction), affected significantly (*p* < 0.05) all the identified volatile compounds. An increase in the storage days decreased the number of terpenoid compounds. A total number of 10, 11, 3, 2 and 1 terpenoid compounds were detected on days 0, 3, 7, 14, and 21, respectively. However, the combinations of HPP and partial substitution of NaCl with KCl at 50, 25 and 0% increased the concentration at day 7 compared to control (Figure 3C). Compared to the control, the HPP-treated samples with partial replacement of NaCl with KCl at 50, 25 and 0% increased the concentration of terpenoids by 48.48, 35.26 and 14.62%, respectively. Similarly, a total number of 5, 4, 2, 0, 1 ester compounds were detected on days 0, 3, 7, 14 and 21, respectively. Compared to the control, the HPP-treated samples with partial substitution of KCl for NaCl at 50, 25, and 0% increased the concentration of esters by 51.94, 30.28, and 23.93%, respectively. However, the pressureless treated samples with partial substitution of NaCl by KCl at 50 and 25% also increased the concentration of esters by 60.17 and 10.23% compared to the control (Figure 3E). In addition, a total number of 4, 5 and 5 acids were detected on days 0, 3 and 7, respectively. However, no acid compounds were detected on days 14 and 21 in the samples treated with or without pressure with partial substitution of NaCl by KCl across the salts levels (Figure 3F). Compared to the control on day 7, the HPP-treated samples with partial substitution of NaCl by KCl at 50, 25, and 0% increased the concentration of acidic compounds by 48.60, 38.84, and 26.59%, respectively. The pressureless treated samples with partial substitution of NaCl by KCl at 50 and 25% also increased the concentration of acidic compounds by 18.16 and 1.83% compared to the control (Figure 3F). Most of the compounds concentration in this current study increased during storage which could be due to lipid oxidation. The increase being dependent on the efficiency in excluding air (48). An expected result has been that, either with or without substitution of NaCl with KCl, HPP caused an increase of many volatile compounds (Figure 3H), some of them coming from amino acid catabolism such as the branched-chain aldehydes, and many others from lipid oxidation, such as linear aldehydes, alcohols, esters and ketones. The esters formed might be either synthesized from free fatty acids and alcohols (esterification) or by transesterification reaction (alcoholysis) of fatty acids of the triglycerides and ethanol (49). Lipid oxidation of sausage can be affected by some external technological factors, such as processing temperature, time and NaCl content, however, the mechanism of flavor formation in sausage still needs to be elucidated. NaCl is an important component in meat processing, especially for cured meat. It endows good flavor to products, inhibits the growth of microorganisms, promotes the lipid oxidation, and affects the activity of lipoxygenase and lipid hydrolytic enzymes (50). Research studying the effect of different NaCl contents on lipid hydrolysis suggested that the increase of NaCl content can promote lipid hydrolysis.

### Effects of Partial Substitution of Sodium Chloride With Potassium Chloride and High Pressure Processing on Phenol and Alkane Compounds

Our findings indicate the changes in phenols and alkanes were non-linear with salt levels, storage time and HPP. The increased in volatile compositions by pressure applied was caused by lipid oxidation induced by HP as initiated by the presence of low-molecular-weight iron compounds and myoglobin, hemoglobin, and ferritin in the meat (51). A total number of 12, 7, 9, 9, and 9 phenolic compounds were significantly affected by HPP and partial substitution of NaCl by KCl on days 0, 3, 7, 14 and 21, respectively. Compared to the control, the HPP treated samples with partial substitution of NaCl with KCl at 50, 25 and 0% increased the concentration of phenolic compounds by 79.05, 55.43 and 17.72%, respectively on day 0. The samples treated without pressure with partial substitution of NaCl by KCl at 50 and 25% also increased the concentration of phenolic compounds by 69.97 and 52.49% compared to the control (Figure 3G) on day 0. Likewise, a total number of 11, 14, 10, 5 and 7 alkane compounds were detected on days 0, 3, 7, 14 and 21, respectively. Compared to the control on day 0, all the HPP-treated samples with partial substitution of NaCl by KCl at 50, 25 and 0% decreased the concentration of alkane compounds by 17.16, 94.64 and 62.67%, respectively. The samples treated without pressure with partial substitution of NaCl by KCl at 50 and 25% also decreased the concentration of alkane compounds by 47.03 and 92.85% compared to the control (Figure 3H). Hexanal is often used as a marker compound for lipid oxidation in meats (52). Generally, the production of most phenols maybe derived from carbohydrate metabolism and lipid β-oxidation and amino acid catabolism (53) while acids are formed through Strecker degradation of amino acids in the Maillard reaction and lipid oxidation (44, 54). The explanation might be that the enzymatic catalyzed reactions for the formation of these volatiles from the free amino acid are limited (55) or by the lower pH that influenced the adsorption capacity of proteins and longer hydrophobic chain that increases the affinity for proteins (56).

### Odor Activity Value

**FIGURE 4:**
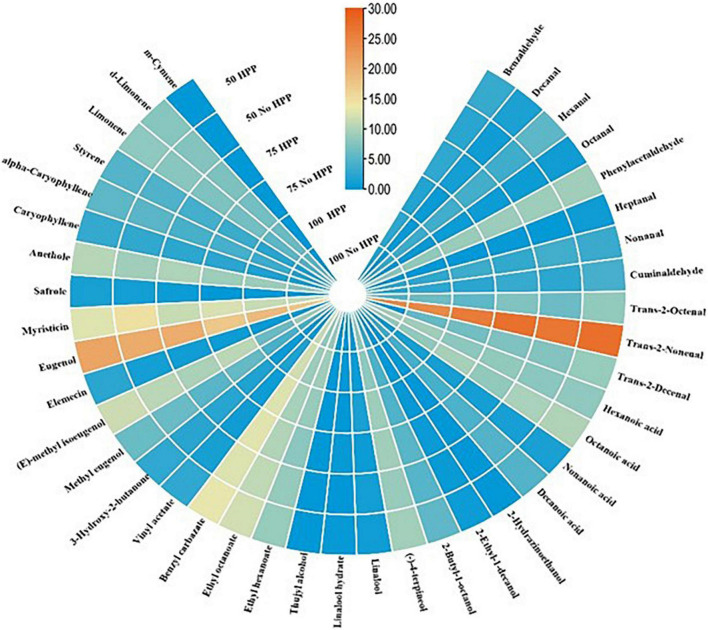
Heat map of odor activity values (OAVs) of volatile compounds in sausage stored at 0 day. The color scale indicates the intensity of the OAV.

**FIGURE 5:**
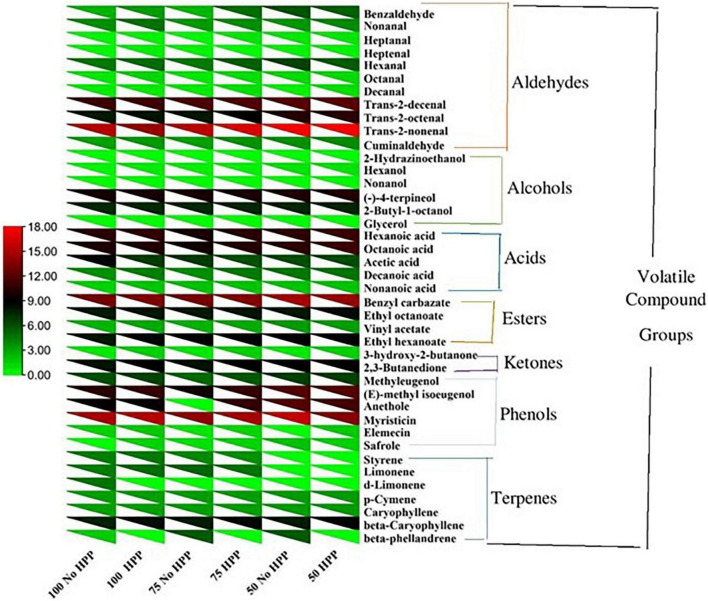
Heat map of odor activity values (OAVs) of volatile compounds in sausage stored at 3 days. The color scale indicates the intensity of the OAV.

**FIGURE 6:**
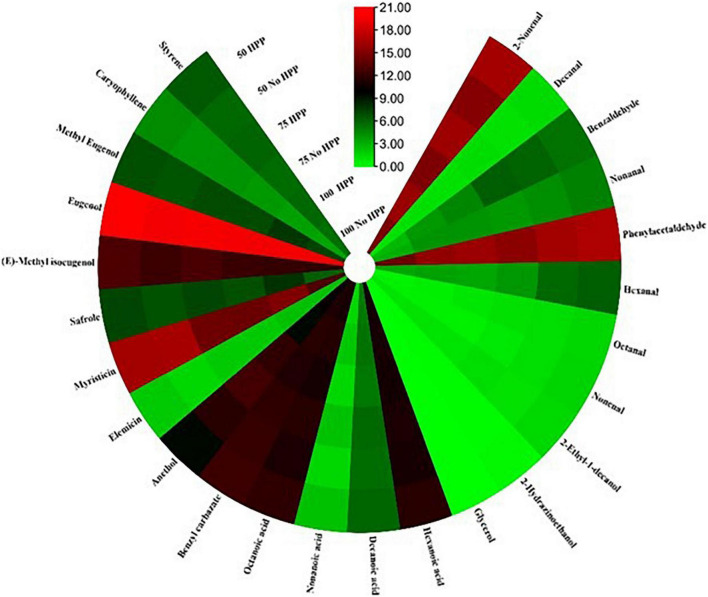
Heat map of odor activity values (OAVs) of volatile compounds in sausage stored at 7 days. The color scale indicates the intensity of the OAV.

**FIGURE 7:**
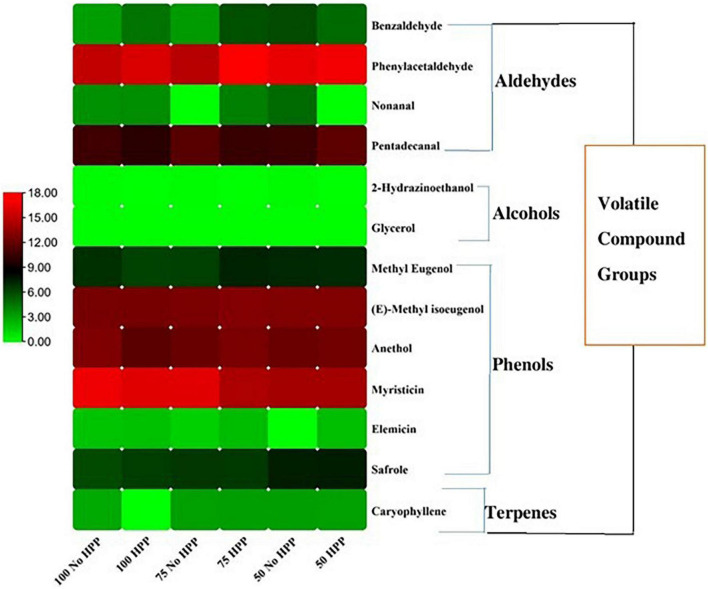
Heat map of odor activity values (OAVs) of volatile compounds in sausage stored at 14 days. The color scale indicates the intensity of the OAV.

**FIGURE 8:**
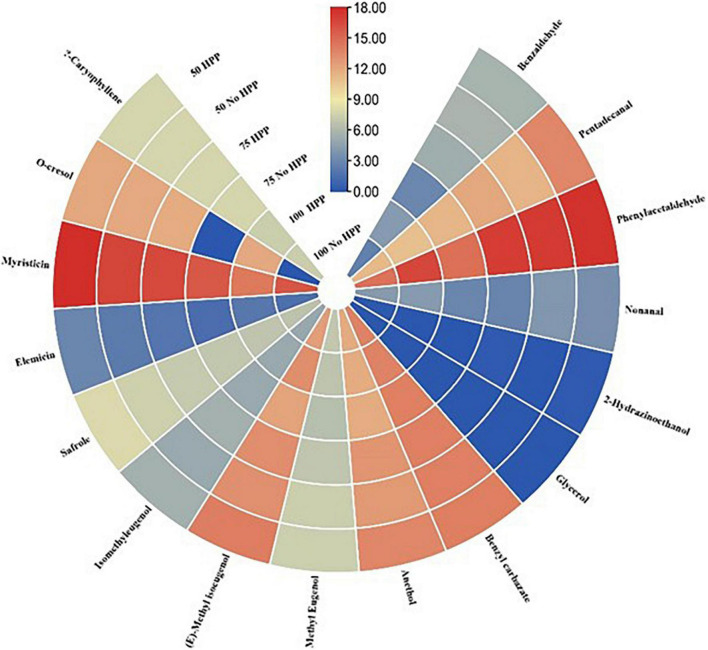
Heat map of odor activity values (OAVs) of volatile compounds in sausage stored at 21 days. The color scale indicates the intensity of the OAV.

With regards to the detection thresholds of the odor-active compounds quantified, odor-activity values (OAV) of the compounds with OAV of 1 or higher are the compounds which contributed most to the aroma of the sausage samples. On day 0, a heat map of 40 volatile compounds with significant differences across the six treatments was observed. Compounds having an OAV > 1 are thought to be the main flavor producers in samples. However, high OAVs (OAV > 1) were obtained after partial substitution of NaCl with KCl at 25 and 50% with HPP treatment compared to the samples not treated with HPP. Compounds such as; trans-2-nonenal, phenylacetaldehyde, trans-2-decenal, hexanoic acid, octanoic acid, myristicin, eugenol, (E)-methyl isoeugenol, ethyl hexanoate, ethyl octanoate, (-)-4-terpineol and benzyl carbazate had an OAV > 1 as compared to the other compounds with an OAV < 1 (Figure 4). On day 3, a heat map of 41 volatile compounds with significant differences across the six treatments was observed. The high OAVs (OAV > 1) were obtained after partial substitution of NaCl with KCl at 25 and 50% with HPP treatment compared to the samples not treated with HPP. Compounds such as; trans-2-nonenal, trans-2-octenal, trans-2-decenal, 2-butyl-1-octanol, 2,3-butanedione, anethole, beta-caryophyllene, hexanoic acid, octanoic acid, myristicin, (E)-methyl isoeugenol, ethyl hexanoate, ethyl octanoate, (-)-4-terpineol and benzyl carbazate had an OAV > 1 as compared to the other compounds with an OAV < 1 (Figure 5). On day 7, a heat map of 25 volatile compounds with significant differences across the six treatments was observed. The high OAVs (OAV > 1) were obtained after partial substitution of NaCl with KCl at 25 and 50% with HPP treatment compared to the samples not treated with HPP. Compounds such as; 2-nonenal, phenylacetaldehyde, eugenol, anethole, hexanoic acid, octanoic acid, myristicin, (E)-methyl isoeugenol, and benzyl carbazate had an OAV > 1 as compared to the other compounds with an OAV < 1 (Figure 6). On day 14, a heat map of 13 volatile compounds with significant differences across the six treatments was observed. The high OAVs (OAV > 1) were obtained after partial substitution of NaCl with KCl at 25 and 50% with HPP treatment compared to the samples not treated with HPP. Compounds such as; phenylacetaldehyde, pentadecanal, methyl-eugenol, anethole, myristicin and (E)-methyl isoeugenol had an OAV > 1 as compared to the other compounds with an OAV < 1 (Figure 7). On day 21, a heat map of 16 volatile compounds with significant differences across the six treatments was observed. Compounds having an OAV > 1 are thought to be the main flavor producers in samples. However, high OAVs (OAV > 1) were obtained after partial substitution of NaCl with KCl at 25 and 50% with HPP treatment compared to the samples not treated with HPP. Compounds such as; pentadecanal, benzyl carbazate, anethole, myristicin, o-cresol, phenylacetaldehyde and (E)-methyl isoeugenol had an OAV > 1 as compared to the other compounds with an OAV < 1 (Figure 8). Many of these volatiles have previously been identified in sausages (57, 58).

## Conclusion

The identified volatile compounds decreased on day 21 with increasing duration of storage. The alcohols, aldehydes, alkanes, esters, phenols, and terpenes were the most abundant groups of volatiles in the beef sausage products. Lipid oxidation seemed to be more prominent in volatile compositions than the amino acid catabolism and esterification during the 21 days’ cold storage. Lipid oxidation products remained stable or decreased during storage. The use of the different salts affected both the lipid oxidation and volatile compounds. Lipid oxidation in combination with the enzymatic activities in the beef sausages contributed to a broad composition of interesting aroma compounds in the product. Even with a high content KCl (50%) in this sausage product, the occurrence of lipid oxidation levels of these vacuum-packed beef sausages was lower than expected. This could be due to partial replacement of NaCl in conjunction with HPP which reduced the degree of lipid oxidation during cold storage that had a positive effect on preventing the formation of undesirable flavor in the beef sausage.

## Data Availability Statement

The raw data supporting the conclusions of this article will be made available by the authors, without undue reservation.

## Author Contributions

LZ and TO: conceptualization. TO: methodology, data curation, and writing—original draft preparation. SB: software. LZ, MA, and TO: validation. ZW: formal analysis. YG: investigation. TO and SB: writing—review and editing. MA: visualization. LZ: resources, supervision, project administration, and funding acquisition. All authors have read and agreed to the published version of the manuscript.

## Conflict of Interest

The authors declare that the research was conducted in the absence of any commercial or financial relationships that could be construed as a potential conflict of interest.

## Publisher’s Note

All claims expressed in this article are solely those of the authors and do not necessarily represent those of their affiliated organizations, or those of the publisher, the editors and the reviewers. Any product that may be evaluated in this article, or claim that may be made by its manufacturer, is not guaranteed or endorsed by the publisher.
